# Culturable bioaerosols along an urban waterfront are primarily associated with coarse particles

**DOI:** 10.7717/peerj.2827

**Published:** 2016-12-22

**Authors:** Angel Montero, M. Elias Dueker, Gregory D. O’Mullan

**Affiliations:** 1 School of Earth and Environmental Sciences, Queens College, City University of New York, Flushing, NY, United States; 2 Environmental and Urban Studies; Biology Program, Bard College, Annandale-on-Hudson, NY, United States; 3 Earth and Environmental Sciences, Graduate Center, City University of New York, NY, United States

**Keywords:** Aerosols, Water quality, Air quality, Culturable bacteria, Coarse aerosol, Urban

## Abstract

The source, characteristics and transport of viable microbial aerosols in urban centers are topics of significant environmental and public health concern. Recent studies have identified adjacent waterways, and especially polluted waterways, as an important source of microbial aerosols to urban air. The size of these aerosols influences how far they travel, their resistance to environmental stress, and their inhalation potential. In this study, we utilize a cascade impactor and aerosol particle monitor to characterize the size distribution of particles and culturable bacterial and fungal aerosols along the waterfront of a New York City embayment. We seek to address the potential contribution of bacterial aerosols from local sources and to determine how their number, size distribution, and taxonomic identity are affected by wind speed and wind direction (onshore vs. offshore). Total culturable microbial counts were higher under offshore winds (average of 778 CFU/m^3^ ± 67), with bacteria comprising the majority of colonies (58.5%), as compared to onshore winds (580 CFU/m^3^ ± 110) where fungi were dominant (87.7%). The majority of cultured bacteria and fungi sampled during both offshore winds (88%) and onshore winds (72%) were associated with coarse aerosols (>2.1 µm), indicative of production from local sources. There was a significant correlation (*p* < 0.05) of wind speed with both total and coarse culturable microbial aerosol concentrations. Taxonomic analysis, based on DNA sequencing, showed that Actinobacteria was the dominant phylum among aerosol isolates. In particular, *Streptomyces* and *Bacillus*, both spore forming genera that are often soil-associated, were abundant under both offshore and onshore wind conditions. Comparisons of bacterial communities present in the bioaerosol sequence libraries revealed that particle size played an important role in microbial aerosol taxonomy. Onshore and offshore coarse libraries were found to be most similar. This study demonstrates that the majority of culturable bacterial aerosols along a New York City waterfront were associated with coarse aerosol particles, highlighting the importance of local sources, and that the taxonomy of culturable aerosol bacteria differed by size fraction and wind direction.

## Introduction

The atmosphere is now widely recognized as a habitat containing numerous and diverse microorganisms (Lighthart, 1997; Pashley et al., 2012; Smets et al., 2016). The concentrations of viable microbial aerosols can vary across temporal and spatial scales (Lighthart, 1997). In the open sea, the concentration of viable airborne bacteria may be on the order of hundreds of cells per cubic meter, while in terrestrial environments, concentrations may be an order of magnitude higher (Lighthart, 1997; Shaffer & Lighthart, 1997; Burrows et al., 2009). Urban coastal environments, like New York City, are unique in that they are located at the interface between land and sea and are heavily exposed to anthropogenic contamination. Depending on wind direction, the flux of bacteria in coastal environments may vary by orders of magnitude (Lighthart, 1997). Bacteria and fungi can be a source of disease and allergens, making their transport through urban environments an area of special concern (Smets et al., 2016). Information about the sources and size distribution of culturable bacteria and fungi aerosols remain as gaps in our understanding of urban microbiology.

An important component of bioaerosols is their aerodynamic size, which determines atmospheric residence time and transport distance. Aerosols with aerodynamic sizes in the order of 1 mm have residence times from seconds to minutes and generally are deposited close to their original source, while those having diameters of 1 µm can have residence times from days to months and reach worldwide distribution (Seinfeld & Pandis, 1998). Bacteria are often associated with larger particles (Lighthart, 2000) and bigger particle sizes can promote higher bacterial survival rates in air (Lighthart & Shaffer, 1997). In marine systems, bacterial cells are strongly associated with sticky hydrated gels which may provide protection from environmental stressors (Blanchard & Syzdek, 1982; Aller et al., 2005). Therefore, bioaerosols associated with coarse particles may be more likely to be locally produced, due to transport range constraints, and more likely to be viable due to stress resistance.

While bioaerosol concentrations are not a well established component of air quality, airborne bacteria and fungi can be important sources of allergens (Yamamoto et al., 2012) and viable pathogens transmitted by air can pose an infection health risk. Outbreaks of foot and mouth disease and Q fever in England have been linked to the aerosolization of pathogens originating from diseased livestock (Donaldson & Alexandersen, 2002; Smith et al., 1993; Hawker et al., 1998). The contamination of the river Taff by *Mycobacterium avium paratuberculosis* has been associated with increased incidence of Crohn’s disease in areas downwind of the river (Pickup et al., 2005). Dust carried to the Caribbean by an African dust storm was significantly associated with increased cases of pediatric asthma (Gyan et al., 2005). Recently, Legionella occurrences in New York City have prompted preventative legislation and alerted the public to the potential of airborne disease transmission in a heavily urbanized environment (https://www.health.ny.gov/diseases/communicable/legionellosis/). Aerosol particle size influences the probability of inhalation or ingestion and the most likely zone of deposition in the human body (Lee et al., 2010), altering potential health relevance.

Wind speed can be positively correlated with airborne microbial concentrations (Lighthart, 1997) and active water surfaces enhance the delivery of marine aerosol into the atmosphere (Aller et al., 2005; Dueker et al., 2011; Dueker et al., 2012a). The disturbance of water surfaces can occur in multiple ways and has relevance to aerosol dynamics. For example, surface aeration of an EPA-designated Superfund waterway in Queens, NY led to elevated bacterial concentrations immediately adjacent to the aeration site (Dueker & O’Mullan, 2014). Similarly, wind speeds over 4 m s^−1^ promote the formation of whitecaps, which consequently release marine aerosols into the atmosphere. In aquatic environments, bursting bubbles deliver the majority of aerosols to the atmosphere (Blanchard & Syzdek, 1982). During higher wind gusts, large aerosols can be transported further inland than during lower wind conditions (Seinfeld & Pandis, 1998). Therefore, wind speed is important in both altering the mechanisms of aerosol production and transport, with the potential to significantly modify microbial airborne communities.

Studies of viable aerosol bacteria in diverse environments show communities dominated by spore formers, which are well adapted to survival in the open atmosphere (Urbano et al., 2011) and may be transported long distances (Shaffer & Lighthart, 1997). However, other studies of airborne bacterial communities suggest that bioaerosol communities often resemble local emission sources. For example, (Carducci, Arrighi & Ruschi, 1995) found bacterial aerosols associated with sewage in close proximity to an activated sludge plant. Aerosol sampling above a New York City aquatic Superfund site detected evidence that the taxonomic identity of bacterial aerosols was influenced by local pollution (Dueker et al., 2012b). Few have studied whether taxonomy changes with aerosol size fraction and wind direction, but these would be reasonable expectations. Knowledge of taxonomy can also help elucidate whether these microbes come from local or regional sources.

Understanding the atmospheric exchange of viable microbes between aquatic and terrestrial environments is an understudied yet important component of microbial ecology and applied environmental microbiology, especially in the urban environment. Directly measuring the concentration and size distribution of culturable bacterial aerosols in the coastal environment is a practical way to constrain the transport potential of viable bacteria from terrestrial and aquatic sources. Although prior studies have enumerated culturable bioaerosols from coastal urban regions, few have simultaneously investigated their size distributions or changes in taxonomic identity with size fraction. In order to address this gap, this study aims to answer several questions pertaining to the local connection between microbial communities in water and air and the characteristics of the bioaerosols present along a New York City waterfront. The goals of this study were to: (1) determine the size distribution of both aerosol particles and microbial aerosols when wind is over land and water; (2) examine the influence of wind speed on fine and coarse bioaerosols; and (3) compare the taxonomic identity of bacteria associated with coarse and fine aerosols, as well as the local water surface. We hypothesized that culturable microbial aerosols would be primarily associated with the coarse fraction; that culturable aerosols would increase with elevated wind speeds, and that taxonomic identity would vary with wind direction and between fine and coarse fractions. The dominance of coarse microbial aerosols would support the importance of local sources and small scale microbiological coupling of water, land, and air.

## Materials & Methods

### Sampling location and methods

**Figure 1:**
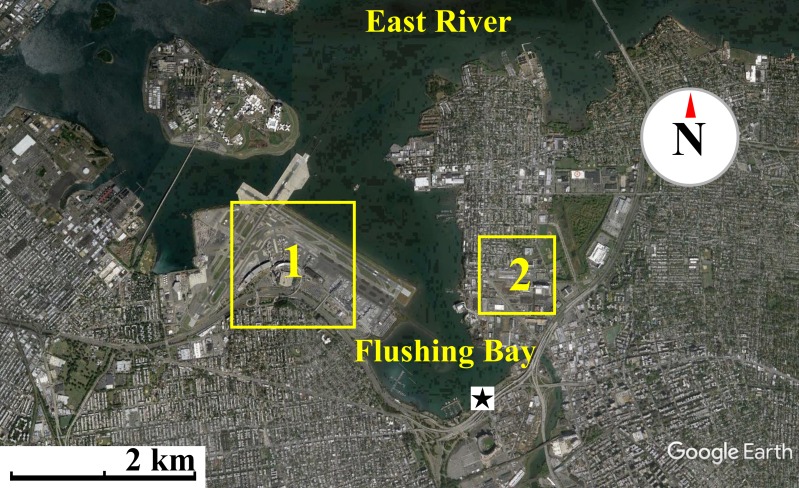
Map of waterfront sampling location. Map of the East River, Flushing Bay & surrounding New York City Area. Sampling site is marked as a star. Flushing Bay is surrounded by La Guardia Airport (1) and College Point (Queens) (2). There is a 550–600 m stretch between College Point and the sampling site and a 1,200–1,300 m stretch between LaGuardia Airport and the sampling site. Map data: Google, sourced from Google Earth, 40°46′28.09″N and 73°51′02.69″W.

Air sampling, at a height of 175 cm from the ground, was conducted during the months of July, August and September of 2015 along the waterfront of Flushing Bay, NY (Fig. 1), an embayment adjacent to the East River in western Long Island Sound. The waterfront is surrounded by industrial and urban residential developments, adjacent highways, LaGuardia International Airport and large recreational areas including Citi Field, the Billie Jean King National Tennis Center and Flushing Meadows Corona Park. A promenade stretches along the shoreline where sampling occurred, and is a site frequently used for recreational activities. Flushing Bay has many combined sewer overflow (CSO) discharges, including one of the largest CSO outfalls in the city, and water quality impairment has resulted in the need for a Long Term Control Plan for sewage pollution (http://www.nyc.gov/html/dep/html/cso_long_term_control_plan/flushing_bay_ltcp.shtml). Enteric bacteria concentrations in Flushing Bay are regularly monitored (http://riverkeeper.org) and previous studies have characterized the microbial composition of the water, including elevated antibiotic resistant bacteria concentrations that are linked to wet weather sewage discharge into the bay (Young, Juhl & O’Mullan, 2013).

Thirty-seven air sampling events were completed over fourteen distinct days, with 23 of the events occurring during onshore winds, on seven unique days, and the remainder completed during offshore wind conditions. Measurements for meteorological conditions, particle concentrations and culturable bioaerosols were conducted in parallel. The start time, duration and volume of air sampled for each event is available in supplemental material (Data S1), along with raw bioaerosol counts. Total aerosol particle concentrations and size distributions were measured at sixty second intervals using an Aerocet-531S Mass Particle Counter (http://www.metone.com). Local weather conditions were logged in sixty second intervals using a Kestrel 4500 weather meter (http://www.kestrelmeters.com). Reference data for local weather conditions were also obtained through the local LaGuardia Airport (KLGA) Weather Underground station (http://www.wunderground.com).

The phrase “culturable microbial aerosols” in this study refers to culturable bacteria and fungi, and they were sampled using an Andersen six-stage viable cascade impactor (Tisch 1800-x, Andersen Inc) with an air flow rate of 28.3 LPM. The cascade impactor fractionates particles into six size bins according to their aerodynamic diameter. The aerodynamic diameter bins are 0.65–1.1 µm (Stage 6), 1.1–2.1 µm (Stage 5), 2.1–3.3 µm (Stage 4), 3.3–4.7 µm (Stage 3), 4.7–7.0 µm (Stage 2), and >7.0 µm (Stage 1). Impacted particles are retained on the surface of agar plates housed in the interior of each chamber. The agar plates were prepared with 27 mL of R2A media, commonly used in aerosol and surface water studies, including other studies in the New York region and Flushing Bay (Young, Juhl & O’Mullan, 2013; Dueker et al., 2012b). Sampling occurred between late morning and late afternoon. At the end of each sampling event the agar plates were quickly covered and stored in a cool container away from sunlight. To assess potential contamination during field activities and the recovery of the plates, blank plates were transported to the field, exposed to air concurrently with the cascade plates for a period of two seconds and handled in parallel with the experimental plates. Because the size bins from the Aerocet Particle Counter and the Anderson Cascade Impactor were not identical, coarse particles were considered as >1 µm, while coarse bioaerosols were considered as >2.1 µm.

### Microbial enumeration and molecular analysis

Following sampling, the agar plates were immediately transported to the lab, and incubated at a temperature of 27 °C for 72 h. Observable bacterial and fungal colonies were then visually enumerated. The raw colony counts were adjusted using the positive hole correction method (Andersen, 1958). Bacterial colonies were picked using sterile pipette tips into individual wells of 96-well plates, suspended in 30 µL of nuclease-free sterile water (Hyclone Laboratories, Inc. Logan, Utah, USA), lysed by boiling at 95 °C for 5 min, and frozen at −20 °C until subsequent molecular analysis. To determine taxonomic identity of bacterial colonies we utilized the approach of Dueker et al. (2012a); Dueker et al. (2012b) and Young, Juhl & O’Mullan (2013) including PCR amplification of lysed colony suspensions using 16s rRNA gene universal bacterial primers 8F (5′-AGRGTTTGATCCTGGCTCAG-3′), and 1492R (5′-CGGCTACCTTGTTACGACTT-3′) (Teske et al., 2002). Amplification reactions included 35 cycles of denaturation (45 s at 95 °C), annealing (45 s at 55 °C ) and elongation (60 s at 72 °C). PCR products were visualized by electrophoresis to confirm amplification of a single approximately 1,500 base pair product and were then processed for single pass Sanger sequencing, using the same 8F primer, by Eton Bioscience (Union, NJ, USA). Sequence quality control and editing was performed using the Geneious software package, and high quality sequences, including a minimum length of 236 base pairs, were uploaded to the Ribosomal Database Project (RDP) for alignment and taxonomic classification to the level of genera using the RDP naïve Bayesian rRNA classifier.

To compare bioaerosol taxonomy with that of local water sources, bacterial 16S rRNA sequences previously obtained from Flushing Bay (Young, Juhl & O’Mullan, 2013), also during the summer season, were assembled into a surface water sequence library for comparison. After quality control filtering removed low quality sequences, a total of 266 16S rRNA sequences were obtained from cascade aerosol isolates and an additional 117 sequences from Flushing Bay surface water isolates. From these sequences three clone libraries were assembled for aerosol sequences, grouped by the wind direction (onshore or offshore) and the size fraction (coarse or fine) where these isolates were observed. Sequences were not obtained from offshore fine isolates. The libraries were designated as onshore fine (*n* = 56); onshore coarse (*n* = 126); offshore coarse (*n* = 84); and surface water (*n* = 117). DNA sequences from bioaerosol isolates have been submitted to the NCBI Genbank database using accession numbers KX959694 –KX959959.

### Statistical analysis

Mann–Whitney nonparametric tests were performed to assess significant differences between the following groups: (1) total fine (<2.1 µm) and total coarse (>21 µm) microbial aerosols; (2) total coarse microbial aerosols under low (<4.0 m s^−1^) and high (>4.0 m s^−1^) wind conditions; and (3) total microbial aerosols (coarse + fine) over high and low wind conditions. To test for significant correlations between culturable microbial aerosols, wind speed, and total particle concentrations, Spearman’s coefficient was used. A Chi-squared test was performed on the distribution of microbial cells over the six cascade aerodynamic size fractions to determine if there was a significant change in size distribution over higher and lower wind speeds. These statistical tests were performed using R statistical software (R Core Team, 2015). For taxonomic analyses, significant differences between sequence libraries were determined using Ribosomal Database Project’s (RDP) Library Compare algorithm (Wang et al., 2007).

## Results

### Aerosol particle size distributions

**Figure 2:**
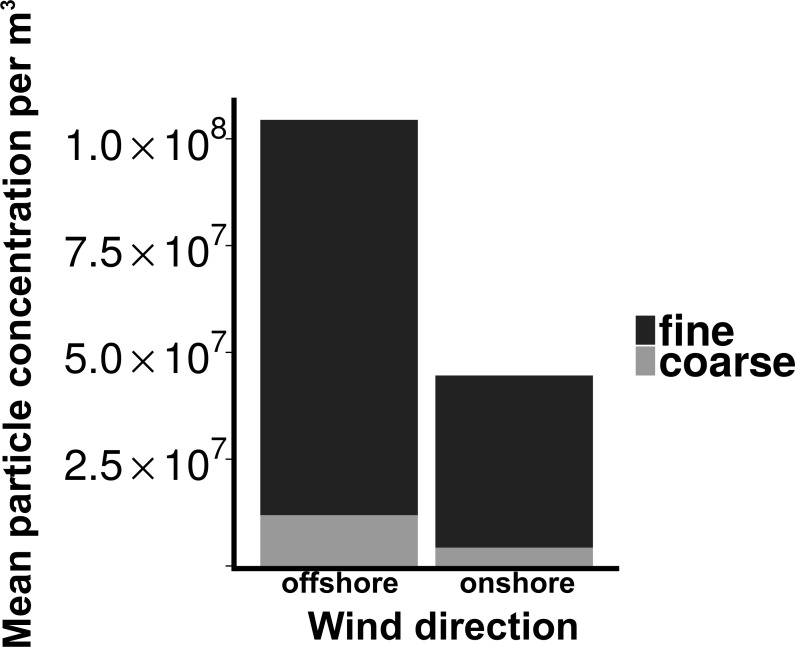
Particle concentrations by wind direction. Mean fine and coarse aerosol particle concentrations for all sampling events. *X*-axis denotes the wind direction during sampling offshore (wind coming over land from the southeast) and onshore (wind coming over water from the northeast). Aerosol particles are characterized as coarse (>1.0 um in diameter) or fine (<1.0 um in diameter).

**Figure 3:**
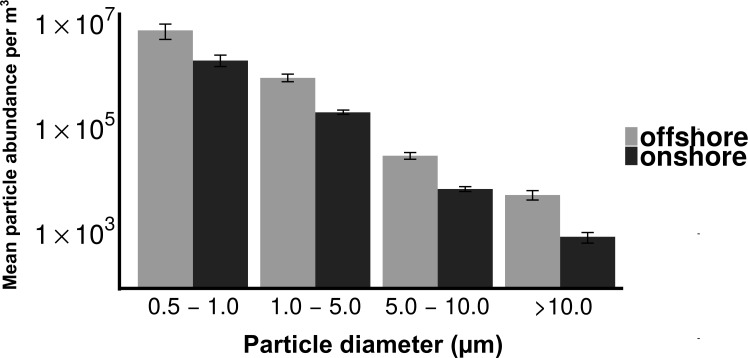
Distribution of particle size fractions by wind direction. Particle size distribution of offshore (top) and onshore (top) aerosols binned into four size fractions. *X*-axis is the particle size bin ranging from particles 0.5–1.0 um to particles >10 um. *Y* axis is the total number of particles measured in each size bin. Error bars represent standard error.

Total particle concentrations during offshore wind conditions (mean = 7.46 × 10^6^m^−3^ ± 2.17 × 10^6^) were approximately four times higher than during onshore wind conditions (mean =1.94 × 10^6^m^−3^ ± 4.52 × 10^5^), with the majority of aerosol particles in the fine fraction (88.6% for offshore, 90.4% for onshore) (Fig. 2). Despite increased concentrations in the offshore wind conditions, both onshore and offshore winds resulted in similar aerosol particle size distributions (Fig. 3).

**Figure 4:**
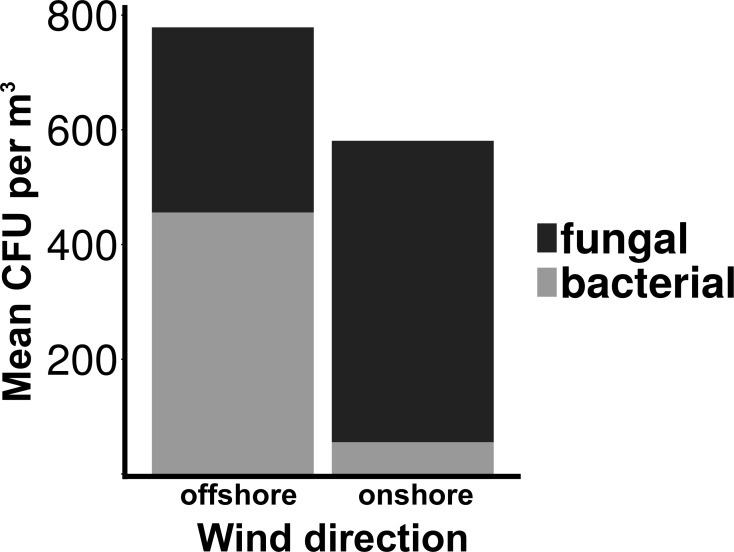
Total culturable aerosols. Mean total colony counts for fungal and bacterial aerosols captured by the Cascade sampler. *X*-axis denotes the wind direction during sampling offshore and onshore. *Y* axis is the number of colony forming units (CFU) per cubic meter.

**Figure 5:**
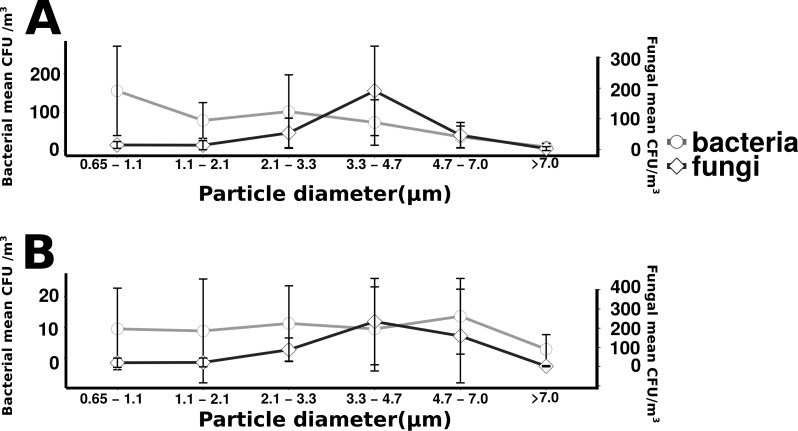
Microbial aerosol size distribution. Size distribution of bacterial and fungal colonies binned by size fraction. The *y*-axis on the left indicates bacterial means, the right axis indicates fungal means. Error bars represent the standard error of the mean. Top plot (A) shows the offshore average number of bacteria and fungi, and the bottom plot (B) corresponds to the onshore average bacteria and fungi.

Total culturable microbial aerosol counts were higher under offshore winds (mean = 778 CFU m^−3^ ± 66.7), with bacteria comprising the majority of colonies (58.5%), as compared to onshore winds (580 CFU m^−3^ ± 110) where fungi were dominant (91.1%) (Fig. 4). Bacterial concentrations were significantly greater during offshore winds (Mann–Whitney, *p* < 0.001), but fungal concentrations did not significantly differ with changing wind direction (Mann–Whitney, *p* = 0.56). The majority of bacterial colonies (90% during offshore, 68% during onshore) were found in the coarse fraction (>2.1 µm) (Fig. 5). Similarly, the majority of fungal colonies were in the coarse fraction (85% during offshore, 70% during onshore) but with a more distinct peak of fungal abundance in Stage 4 (aerodynamic sizes 2.1–3.3 µm).

### Wind speed

**Figure 6:**
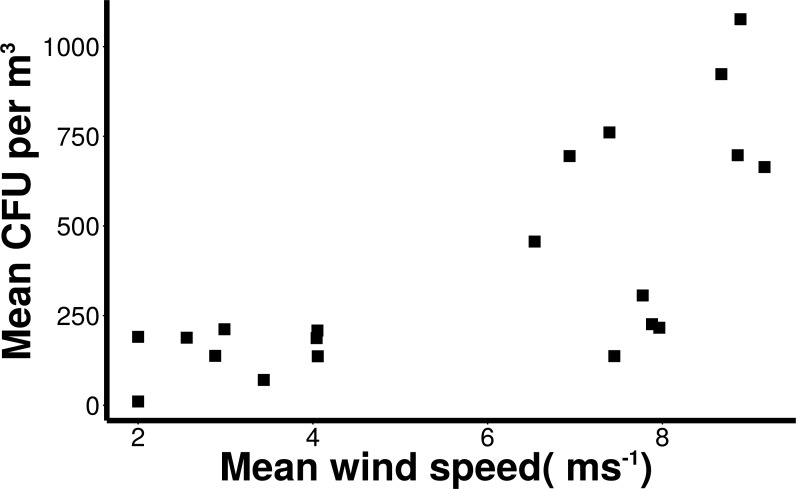
Increasing microbial aerosols with increasing onshore wind speed. Positive correlation (Spearman, *r* = 0.73 and *p* < 0.001) of number of coarse microbial aerosols captured during onshore winds (bacteria and fungi, aerodynamic size > 2.1 um) and mean wind speed.

Wind conditions were found to be important to the distribution and abundance of culturable microbial aerosols. Wind speed and total coarse bioaerosols measured during onshore winds were significantly correlated (Spearman, *r* = 0.73 and *p* < 0.001) (Fig. 6). Under high onshore wind conditions (>4 ms^−1^) , the mean total (fine + coarse) microbial concentration in air was 900 CFU m^−3^ ± 180 (Fig. 6). It was significantly higher than microbial concentrations observed during lower wind conditions (mean = 180 CFU m^−3^ ± 27.6; Mann–Whitney, *p* < 0.001). The size distribution of culturable microbes significantly varied under low and high winds (Chi-squared, *p* < 0.05) for offshore bacteria, offshore fungi, and onshore fungi, with the percentage of fine aerosols increasing under high winds.

### Taxonomic identification

**Figure 7:**
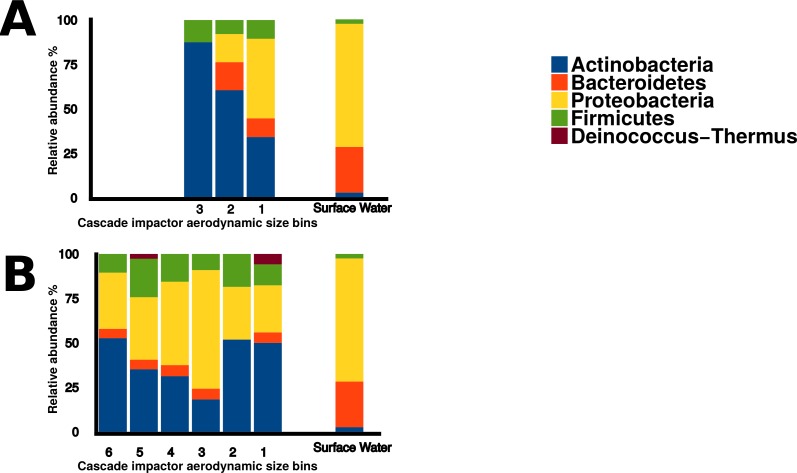
Bacterial aerosol phyla by size fraction and wind direction. Comparison of bacterial aerosol phyla between cascade impactor size fractions. (A) corresponds to offshore coarse library (stages 1–3, >3.3 um). (B) corresponds to onshore fine (stages 6 and 5, <2.1 um) and onshore coarse (stages 4–1, >2.1  um).

**Figure 8:**
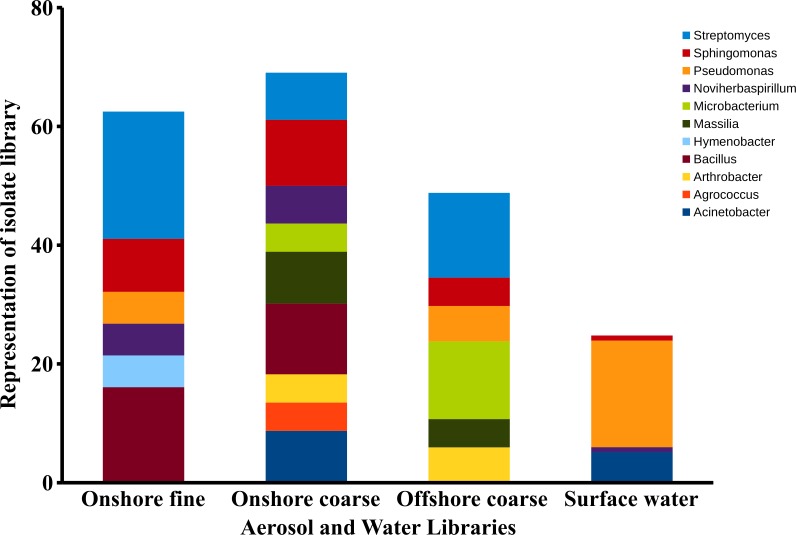
Aerosol isolate genera. Dominant genera in the onshore fine, onshore coarse and offshore coarse isolate libraries. Dominant genera were defined as genera representing at least 4% of the sequences in a single isolate library. The figure represents the percent of sequences classified to these dominant genera.

Sequences within the three aerosol libraries were taxonomically identified and represent 49 genera, with 24 genera occurring as singletons (Table S1). The majority of genera belonged to the Actinobacteria, Proteobacteria, Firmicutes, and Bacteroidetes phyla (Fig. 7) with an additional three sequences in the onshore coarse and fine libraries belonging to Deinococcus–Thermus. Actinobacteria and Proteobacteria were found to be the two most abundant phyla present in all three aerosol libraries (Fig. 7). The most common genera shared by all three aerosol libraries were *Streptomyces*, *Bacillus*, *Sphingomonas*, *Microbacterium*, and *Massilia*, (Fig. 8).

There were significant differences in surface water and aerosol taxonomy. 10% of the surface water isolates were classified as *Flavobacterium*, a genera that was not detected in the aerosol library. *Pseudomonas* comprised 18% of the surface water isolates, yet only comprised 2 to 6 % of the isolates in each of the aerosol libraries. Pairwise comparison of the aerosol and water libraries using the thetayc dissimilarity index (Yue & Clayton, 2005) showed that the three aerosol libraries were more similar to each other than to surface water. The onshore coarse and offshore coarse libraries showed the greatest similarity (thetayc index = 0.375) (Fig. S1).

In the onshore fine aerosol library, *Streptomyces* (21.4%) and *Bacillus* (16.1%) were abundant but were not found in surface waters. In the onshore coarse aerosol library, *Bacillus* (12.0%), *Sphingomonas* (11.1%), and *Acinetobacter* (8.7%) were the most abundant genera and *Sphingomonas*, *Streptomyce*s, and *Bacillus* were significantly more abundant in the onshore coarse library than in surface waters (*p* < 0.05). For the offshore coarse library, *Streptomyces* (14.3%) and *Microbacterium* (13.1%) the most common genera, which were significantly more abundant than in surface water (*p* < 0.05).

## Discussion

For onshore and offshore aerosols, the majority of the enumerated bacteria were associated with particles greater than 2.1 µm (Fig. 5), indicating that airborne culturable microbes are primarily associated with coarse particles. The dominance of culturable bacteria within the coarse fraction is strong evidence that most culturable bacterial aerosols are not free-living, but instead associated with water droplets, organic debris, hydrated gels or other cell-aggregating materials as has been observed in prior studies (Aller et al., 2005; Maron et al., 2005). For onshore wind conditions it is likely that bacteria have formed aggregates in association with gelatinous material originating from the surface microlayer (Aller et al., 2005). There may also be large numbers of non-particle associated bacteria transported in smaller size fractions but, if present, few were culturable. The decision to utilize cultivation techniques has limitations for representing total microbial diversity, as has been discussed in prior studies (Dueker & O’Mullan, 2014; Young, Juhl & O’Mullan, 2013) but provides the important advantage of knowing that the enumerated cells are viable. Not all viable cells can be cultured, especially with a single media type incubated at a single temperature, so the methodologies in this study enumerate only a subset of total viable cells. In addition, not all culturable isolates can be amplified with universal primers and, therefore, not all enumerated isolates can be taxonomically identified. However, the methodologies used are held constant across the study and allow a relative comparison of culturable cells. Cultivation based approaches, unlike cultivation independent sequencing of environmental DNA, also have the advantage that all enumerated cells are known to be viable. Viability plays a major role in the biogeochemical and public health implications for microbial aerosol production and transport, and is expected to differ across aerosol size fractions, which is important to this study.

Although fungal concentrations did not significantly change, culturable bacterial aerosols were ten times higher during offshore than in onshore winds (Fig. 4). Wind direction is an important factor modulating urban coastal air quality by changing the sources of aerosol production, as demonstrated by the significant differences in the number of culturable bacterial aerosols resulting from onshore versus offshore wind conditions along the Flushing Bay waterfront. Although urban waterways have been demonstrated by prior studies (Dueker et al., 2012b; Dueker & O’Mullan, 2014) to be a source of aerosols, the concentrations of both particles and culturable microbial aerosols in this study were higher with offshore winds, compared to onshore winds (Fig. 4), a finding that is generally consistent with expectations from the prior literature (Shaffer & Lighthart, 1997). The ratio of bacteria to fungi also differed for onshore versus offshore winds, with higher concentrations of bacteria and higher ratios of bacteria to fungi with offshore winds coming from the urban terrestrial environment. These differences with wind direction reinforce the need to understand the diverse terrestrial and aquatic sources contributing to aerosol formation in this urban environment.

Despite the differences in aerosol concentration, particle size distributions were quite similar for onshore and offshore winds (Fig. 3), with each dominated by fine particles (>85%). Given the dominance of small particles, many aerosols in this coastal urban air may have originated from sources kilometers to hundreds of kilometers from the sampling location (Fig. S2) (Seinfeld & Pandis, 1998). In contrast, culturable microbial aerosols were almost entirely associated with the coarse fraction, suggesting a more local production source, and the potential importance of protection from UV, desiccation and other stressors that are expected with larger particles and shorter transport times (Kundsin, 1968). The differing size distributions of particles and culturable microbes is also important from the perspective of inhalation and health outcomes. Most urban air quality studies consider fine and coarse particulate material but they rarely measure microbial aerosols directly. A viable cell is likely to be deposited in the lung if it is within the range of 1–5 µm (Lee et al., 2010). The majority of the bacterial aerosols sampled in this study were present in the coarse fraction (2.5–7 µm), and many could be deposited in the lung. Consequently these culturable aerosols could be expected to have an impact on air quality from a health perspective.

The persistence of fungal spores, adapted for long range transport, is thought to contribute to their abundance in air (Yamamoto et al., 2012). Fungi can be an important source of allergens and other health risks that may affect individuals regardless of initial health status. The aerodynamic size of the fungal spore inherently influences the health risk associated with the spore (Cho et al., 2005). In contrast to the wide distribution of bacterial aerosols within coarse size fractions, culturable fungi were found to have a distinct peak (aerodynamic sizes 3.3–4.7 µm), a pattern that has been reported in other studies (Alghamdi et al., 2014; Li et al., 2011). A recent study in nearby New Haven, Connecticut found fungal size distribution to have little variability by season, with a peak size of approximately 2–5 µm, identified primarily as Basidiomycota, while larger size fractions were determined to be primarily Ascomycota (Yamamoto et al., 2012). Fungi in this size range (2–5 µm) are less likely to be aggregate-associated and may be transported longer distances as spores, suggesting regional rather than local influences. The relatively higher fungal concentration of onshore winds (Fig. 4) along with the expectation for longer fungal viability in the atmosphere may suggest the contribution of regional sources kilometers upwind of the sampling site, a reasonable transport scenario for culturable fungi (peaking in 3.3 to 4.7 µm stage) under elevated wind conditions (Fig. S2). At the scale of tens to hundreds of kilometers the “onshore” winds would actually be coming from terrestrial environments, beyond Flushing Bay from the northwest, and “offshore” winds would be coming from a mixture of terrestrial and oceanic environments to the southeast (Fig. 1).

### Wind speed

Wind speed was correlated with total numbers of airborne bacteria and fungi. Regardless of particle size, large winds may transport bacterial and fungal spores kilometers from their source, affecting air quality on a regional or even global scale. Dust from Saharan Desert storms often penetrate Europe, and even cross the Atlantic to deposit in the Caribbean Ocean (Gyan et al., 2005). This has important health and environmental connotations. Strong wind conditions can transport airborne pathogens through large, populated areas. Such was the case during a foot and mouth disease epidemic in England (Hugh-Jones & Wright, 1970). Prior studies involving culturable microbes and meteorological factors have established a positive correlation between wind speed and airborne bacteria concentrations (Lighthart, 1997); however, other studies did not find a correlation between wind speed and culturable bacteria (Li et al., 2011). The association of wind speed and coarse aerosols in this study reinforces the importance of local production sources for this urban waterfront.

### Taxonomy

Actinobacteria and Proteobacteria were the predominant bacterial phyla present in aerosol sequences. Proteobacteria is one of the most diverse phyla among the bacteria and has been found to be common in aerosols across a wide variety of regions and environments (Dueker et al., 2012a). Actinobacteria have been found in cloud water, and urban and rural aerosols (Amato et al., 2005; Maron et al., 2005; Lee et al., 2010; Dueker et al., 2012b), but were not dominant in Flushing Bay surface water samples, where Proteobacteria and Bacteroidetes were the most abundant. Some genera were detected in aerosol samples but were absent in surface water libraries, such as *Streptomyces* and *Bacillus* which are generally associated with soil, and are also capable of forming spores (Nicholson, 2002; Vetsigian, Jajoo & Kishony, 2011). This would enable them to remain viable in the atmosphere, and they may be transported long distances, providing a regional signal disconnected from local water sources.

Many of the genera observed in Flushing Bay air have also been observed in prior aerosol studies. For example, using a similar sampling approach, Wang et al. (2012) found that some of the dominant genera inside cave air included *Micrococcus*, *Sphingomonas*, *Pseudomonas*, and *Bacillus*; which were also dominant in our aerosol samples. Urbano et al. (2011) found a dominance of *Bacillus* in marine aerosol sampled in California. Using culture independent approaches, Amato et al. (2005) found a dominance of *Streptomyces*, *Microbacterium*, *Micrococcus*, *Pseudomonas*, and *Bacillus* in cloud water. The wide spread presence of these bacteria among diverse environments, including urban and rural environments, suggests the importance of cellular adaptations to aerosol formation or atmospheric persistence.

Aerosol particle size may control microbial exposure to environmental conditions. Small particle diameters imply a higher degree of exposure to the harsh conditions of the atmosphere, while bacteria that clump to each other or adhere to larger particles may be better sheltered from these stresses. The observed difference in coarse and fine onshore taxonomy may therefore reflect adaptation to much harsher conditions in the atmosphere. For example, isolates identified as Deinococcus–Thermus, a phyla adapted to tolerate high levels of radiation and desiccation (Battista, Earl & Park, 1999), were found in our aerosol samples.

Although transport models (e.g., Fig. S2) often indicate a range for particles of several hundred kilometers, this may be an overestimate for most bacteria, as the models generally do not account for viability or culturability. There are likely a subset of taxa (e.g., Deinococcus–Thermus) that may remain viable longer and their transport may be more constrained by physical, rather than biological constraints. However, biological response to stress is likely to be a major constraint for most taxa. Viability and culturability are expected to decrease with transport distance, making the actual range of viable bacterial transport much shorter than most models predict. This is consistent with the observed low number of culturable microbes in the fine fraction, which would be expected to travel the longest distances.

## Conclusions

Along an urban waterfront of New York City, the majority of culturable microbial aerosols were associated with coarse particles greater than 2.1 um. Wind direction influenced the taxonomy and concentration of culturable bacteria in the air, while the concentration of fungi did not significantly vary. Onshore wind speed was an important control on the number of culturable total and coarse microbial aerosols, likely due to local production mechanisms from the water surface. In summary, this study highlights the importance of coarse aerosol particles and local production mechanisms as controls on the microbial ecology of aerosols along this urban waterfront.

##  Supplemental Information

10.7717/peerj.2827/supp-1 Table S1 Taxonomic classification of bioaerosol isolates Classification of 16S rRNA sequences obtained from isolates of onshore fine (*n* = 56), onshore coarse (*n* = 126), offshore coarse (*n* = 84), and surface water (*n* = 117) libraries. Classification based on Ribosomal Database Project classifier tool at 95% confidence unless otherwise noted by footnote.

10.7717/peerj.2827/supp-2 Figure S1 Similarity tree, Thetayc, of bioaerosol isolate sequence libraries Thetayc similarity tree of the three aerosol and surface water 16S rRNA libraries: onshore fine (WF), onshore coarse (WC), offshore coarse (LC) and surface water (FBWATER). Thetayc similarity comparison was based on a 97% OTU definition.

10.7717/peerj.2827/supp-3 Figure S2 Particle transport model Estimation of distance traveled by a particle of 3 different sizes and under 3 different wind speeds. Assumptions: (1) Laminar air flow with no turbulence to disrupt the path of the particle; (2) A flat surface with no obstacles; (3) Horizontal velocity of the particle equal to wind speed; and (4) Vertical velocity equal to the terminal velocity of the particle in air.

10.7717/peerj.2827/supp-4 Data S1 Aerosol raw data Aerosol data including: sampling times, volumes, and raw bioaerosol counts.

10.7717/peerj.2827/supp-5 Supplemental Information 1 Fasta file for aerosol isolate sequences Fasta file for 16S rRNA gene sequences from aerosol sequences. Sequences will also be available in the NCBI genbank database using accession numbers KX959694 –KX959959, released October 14th, 2016.

## Additional Information and Declarations

### DNA Deposition

The following information was supplied regarding the deposition of DNA sequences: DNA sequences of the 16S rRNA gene from isolates have been submitted to the NCBI genbank database using accession numbers KX959694 –KX959959.

### Data Availability

The following information was supplied regarding data availability: The raw data has been supplied as Supplementary File.
