# Supplementary material for: Culturable bioaerosols along an urban waterfront are primarily associated with coarse particles
Source: PeerJ. 2016 Dec 22;4:e2827. doi: 10.7717/peerj.2827 (PMC5182991; doi:10.7717/peerj.2827)
Supplement: Table S1 — Classification of 16S rRNA sequences obtained from isolates of onshore fine (n = 56), onshore coarse (n = 126), offshore coarse (n = 84), and surface water (n = 117) libraries. Classification based on Ribosomal Database Project classifier tool at 95% confidence unless otherwise noted by footnote. [file peerj-04-2827-s001.pdf]

Table 1: Classification of 16S rRNA sequences obtained from isolates of onshore fine (n = 56), onshore coarse (n = 126), offshore coarse (n = 84), and surface water (n = 117) libraries. Classification based on Ribosomal Database Project classifier tool at 95% confidence unless otherwise noted by footnote.

| **Phylum** | **Family** | ***Genus*** | **% of Onshore fine** | **% of Onshore coarse** | **% of Offshore coarse** | **% of Surface water** |
|---|---|---|---|---|---|---|
| ***Proteobacteria*** | *Sphingomonadaceae* | *Sphingomonas* | 8.93 | 11.9 | 4.76 | 0.85 |
| | | *Sphingobium* | | | | 5.98[a] |
| | | *Novosphingobium* | 3.57 | 0.79 | | |
| | | *Sphingorhabdus* | | | | 0.85 |
| | *Pseudomonadaceae* | *Pseudomonas* | 5.36 | 2.38 | 5.95 | 18.8[b] |
| | | unclassified | | | | 0.85 |
| | *Oxalobacteraceae* | *Massilia* | 1.79 | 8.73 | 7.14 | |
| | | *Noviherbaspirillum* | 5.36 | 5.56[b] | | 0.85 |
| | | unclassified | | 0.79 | | |
| | *Moraxellaceae* | *Acinetobacter* | 1.79 | 8.73 | 3.57 | 5.13 |
| | | *Psychrobacter* | | | | 4.27 |
| | | *Paraperlucidibaca* | | | | 0.85 |
| | | unclassified | | | 1.19 | |
| | *Campylobacteraceae* | *Arcobacter* | | | | 10.26 |
| | *Comamonadaceae* | *Hydrogenophaga* | | | | 1.71 |
| | | *Acidovorax* | | | | 0.85 |
| | | *Simplicispira* | | | | 0.85 |
| | | *Variovorax* | | 0.79 | | |
| | | unclassified | | | | 1.71 |
| | *Caulobacteraceae* | *Brevundimonas* | 1.79 | 0.79 | 2.38 | 0.85 |
| | | *Caulobacter* | | | | 0.85 |
| | *Acetobacteraceae* | *Roseomonas* | | 0.79 | 3.57 | |
| | *Shewanellaceae* | *Shewanella* | | | | 3.42 |
| | *Aeromonadaceae* | *Aeromonas* | | | | 2.56 |
| | *Methylobacteriaceae* | *Methylobacterium* | 1.79 | 0.79 | | |
| | | *Microvirga* | 1.79 | | | |
| | *Rhizobiaceae* | *Rhizobium* | | | | 1.71 |
| | *Rhodospirillaceae* | *Azospirillum* | | | | 0.85 |
| | | *Skermanella* | 1.79 | | | |
| | *Xanthomonadaceae* | *Stenotrophomonas* | | | | 0.85 |
| | | unclassified | | 0.79 | | |
| | *Chromatiaceae* | *Rheinheimera* | | | | 0.85 |
| | *Enterobacteriaceae* | *Escherichia/Shigella* | | | | 0.85 |
| | *Erythrobacteraceae* | *Erythrobacter* | | | | 0.85 |
| | *Ferrimonadaceae* | unclassified | | | | 0.85 |
| | *Rhodobacteraceae* | unclassified | | | | 0.85 |
| ***Actinobacteria*** | *Microbacteriaceae* | *Microbacterium* | 1.79 | 5.56[b] | 13.1 | |

| | | | | | | |
|---|---|---|---|---|---|---|
| | | *Agrococcus* | | 4.76 | 2.38 | |
| | | *Curtobacterium* | | 1.59 | 2.38 | |
| | | *Amnibacterium* | | 0.79[a] | | |
| | | *Cryobacterium* | | 0.79 | | |
| | | *Labedella* | 1.79 | | | |
| | | *Mycetocola* | | | 1.19 | |
| | | unclassified | | | 1.19 | |
| | *Streptomycetaceae* | *Streptomyces* | 21.43 | 7.94 | 14.29 | |
| | *Micrococcaceae* | *Arthrobacter* | | 4.76 | 5.95 | 0.85 |
| | | *Kocuria* | | 2.38 | 3.57 | |
| | | *Micrococcus* | 1.79 | 1.59 | | |
| | | unclassified | | | 1.19 | |
| | *Pseudonocardiaceae* | *Saccharothrix* | 1.79 | 0.79 | | |
| | | *Amycolatopsis* | 1.79 | | | |
| | | *Lentzea* | | | 1.19[a] | |
| | | *Umezawaea* | 1.79[a] | | | |
| | | unclassified | | | 1.19 | |
| | *Nocardioidaceae* | *Nocardioides* | 1.79 | 0.79 | 1.19 | |
| | | *Aeromicrobium* | 1.79 | | | |
| | *Cellulomonadaceae* | *Cellulomonas* | | 0.79 | | |
| | | unclassified | 1.79 | | | |
| | *Intrasporangiaceae* | *Terrabacter* | 1.79 | 0.79 | | |
| | *Nocardiaceae* | *Rhodococcus* | 1.79 | 0.79 | | |
| | *Promicromonosporaceae* | *Cellulosimicrobium* | | 1.59 | | |
| | *Bogoriellaceae* | *Georgenia* | | | 1.19 | |
| | *Corynebacteriaceae* | *Corynebacterium* | | | 1.19 | |
| | *Dermabacteraceae* | *Brachybacterium* | | | | 0.85 |
| | *Dietziaceae* | *Dietzia* | | 0.79 | | |
| | *Geodermatophilaceae* | *Modestobacter* | | 0.79 | | |
| | *Sanguibacteraceae* | *Sanguibacter* | | | | 0.85 |
| *Bacteroidetes* | *Flavobacteriaceae* | *Flavobacterium* | | | | 14.53 |
| | | *Chryseobacterium* | | | 2.38 | 0.85 |
| | | *Cloacibacterium* | | | | 0.85 |
| | | *Epilithonimonas* | | | | 0.85 |
| | | unclassified | | | 1.19 | 2.56 |
| | *Cytophagaceae* | *Hymenobacter* | 5.36 | 3.17 | | |
| | | *Adhaeribacter* | | | 1.19 | |
| | | *Dyadobacter* | | 0.79 | | |
| | | *Fluviimonas* | | | | 0.85 |
| | | *Pontibacter* | | | 1.19 | |
| | | *Siphonobacter* | | 0.79 | | |
| | *Chitinophagaceae* | *Flavihumibacter* | | | | 0.85 |
| | | unclassified | | | 4.76 | |

| | | | | | | |
|---|---|---|---|---|---|---|
| | *Cyclobacteriaceae* | unclassified | | | | 4.27 |
| | *Sphingobacteriaceae* | *Pedobacter* | | | 1.19 | |
| ***Firmicutes*** | *Bacillaceae* | *Bacillus* | 16.07 | 11.9 | 3.57 | |
| | *Carnobacteriaceae* | *Trichococcus* | | | 1.19 | 1.71 |
| | | *Desemzia* | | 0.79 | | |
| | *Planococcaceae* | *Chryseomicrobium* | | | 2.38 | |
| | | *Planomicrobium* | | | | 0.85 |
| | *Paenibacillaceae* | *Brevibacillus* | | | 1.19 | |
| | | *Paenibacillus* | 1.79 | | | |
| | *Bacillales Incertae Sedis XII* | *Exiguobacterium* | | 0.79 | | |
| ***Deinococcus-Thermus*** | *Deinococcaceae* | *Deinococcus* | 1.79 | 1.59 | | |

[a]Indicates that one of the sequences was classified with 85-95% confidence; [b]indicates that two of the sequences were classified with 85-95% confidence. Any sequence with a confidence level less than 85% for classification at the level of genus is listed as 'unclassified' in the table.
