# Supplementary figures and images for: Culturable bioaerosols along an urban waterfront are primarily associated with coarse particles
Source: PeerJ. 2016 Dec 22;4:e2827. doi: 10.7717/peerj.2827 (PMC5182991; doi:10.7717/peerj.2827)

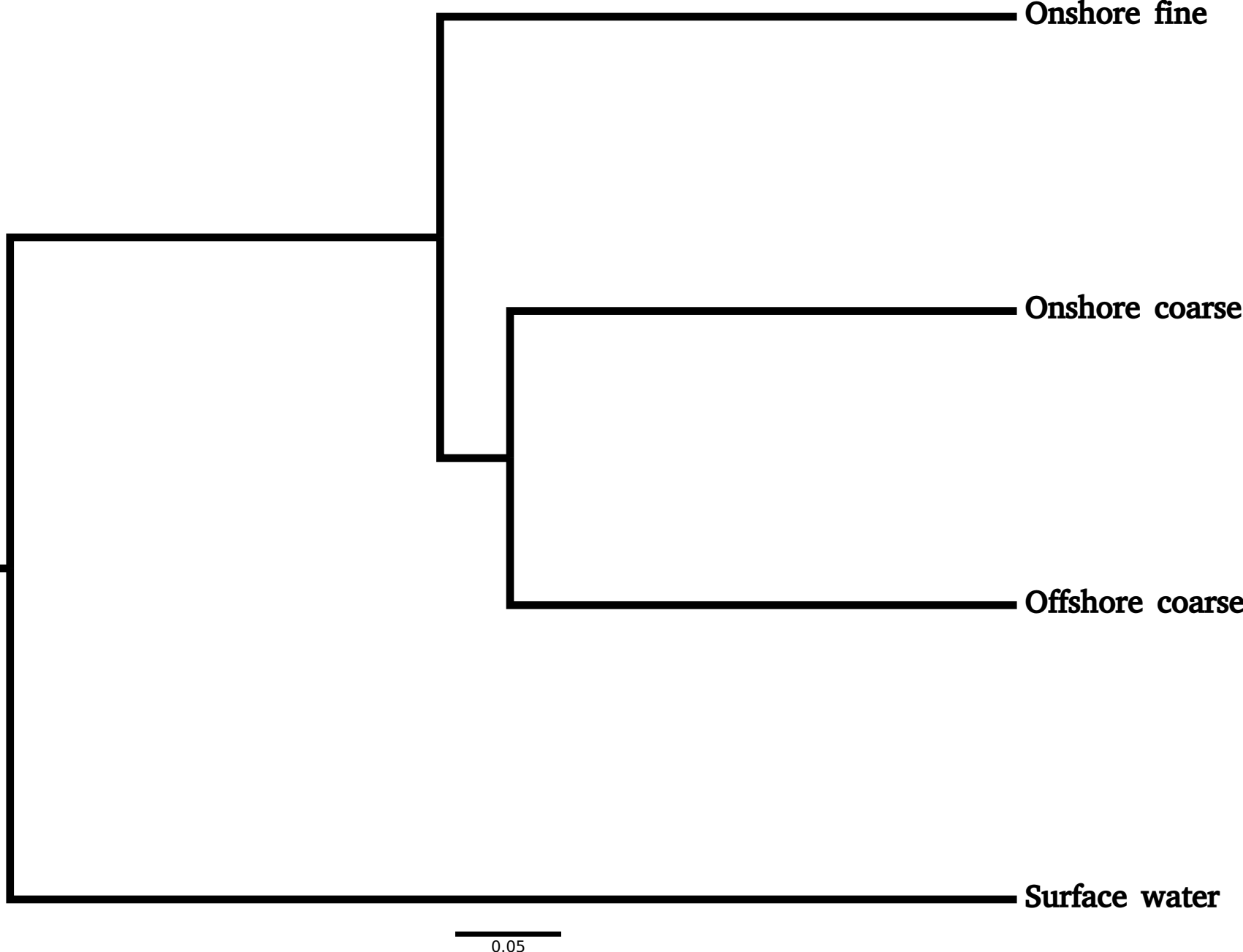

Supplement: Figure S1 — Thetayc similarity tree of the three aerosol and surface water 16S rRNA libraries: onshore fine (WF), onshore coarse (WC), offshore coarse (LC) and surface water (FBWATER). Thetayc similarity comparison was based on a 97% OTU definition. [file peerj-04-2827-s002.pdf]
