# Supplementary material for: Culturable bioaerosols along an urban waterfront are primarily associated with coarse particles
Source: PeerJ. 2016 Dec 22;4:e2827. doi: 10.7717/peerj.2827 (PMC5182991; doi:10.7717/peerj.2827)

Supplemental text and Figure S2 related to particle range estimation:

Given the size of an aerosol particle and wind speed, it is possible to estimate a range of transport for the particle while it remains suspended in the atmosphere. An expression that describes this in air is Stoke's Law (Seinfeld & Pandis , 1998):

$$F_{\mathrm{drag}} = \frac{3\,\pi\mu\,u_{\infty}D_p}{C_c}$$

Where:
μ = viscosity of the fluid
u_{∞} = relative velocity of the particle in the fluid
D_p = particle diameter
C_c = Cunningham Slip Correction Factor

A simple model was constructed to estimate how far a particle, with a known height above the ground, would be carried downwind before impacting the ground. The model applies to a spherical particle with known diameter, a flat horizontal plane, and a fluid medium (air) at standard temperature and pressure. The model assumes laminar flow, zero net external force on the particle, a particle falling down towards the plane with vertical velocity equal to its terminal velocity in air, initial height equal to the height of the Andersen Sampler (175 cm), and horizontal velocity equal to wind speed ($u_{\infty} = 0$).

The formula for determining the terminal velocity of a particle in a fluid medium (Seinfeld & Pandis, 1998) is described as:

$$\nu_{\mathrm{t}} = \frac{1}{18} * \frac{D_p^2\,\rho_p g C_c}{\mu}$$

Where:
ρ_p = density of the particle (g cm^{-3})
g = 981 cm s^{-1}

Using these two formulas under the constraints described, we calculated the potential distance a locally-produced aerosol particle could travel before settling to the ground. The particle transport model figure, below, demonstrates that changes in particle size and wind speed create the potential for aerosol transport distances to vary by orders of magnitudes, from hundreds of meters to hundreds of kilometers. Particle size is an important determinant of transport distance and, even under low wind speed, fine aerosol particles can be transported tens of kilometers. However, under low wind speeds, large particles can be constrained to very local sources (hundreds of meters). Elevated wind speeds can counteract the effect of particle size, with particles as large as ten microns being transported for kilometers under very high wind speeds, which at the scale of an urban environment is the difference between local and regional transport.

Figure legend S2. Estimation of distance traveled by a particle of 3 different sizes and under 3 different wind speeds. Assumptions: 1) Laminar air flow with no turbulence to disrupt the path of the particle; 2) A flat surface with no obstacles; 3) Horizontal velocity of the particle equal to wind speed; and 4) Vertical velocity equal to the terminal velocity of the particle in air.

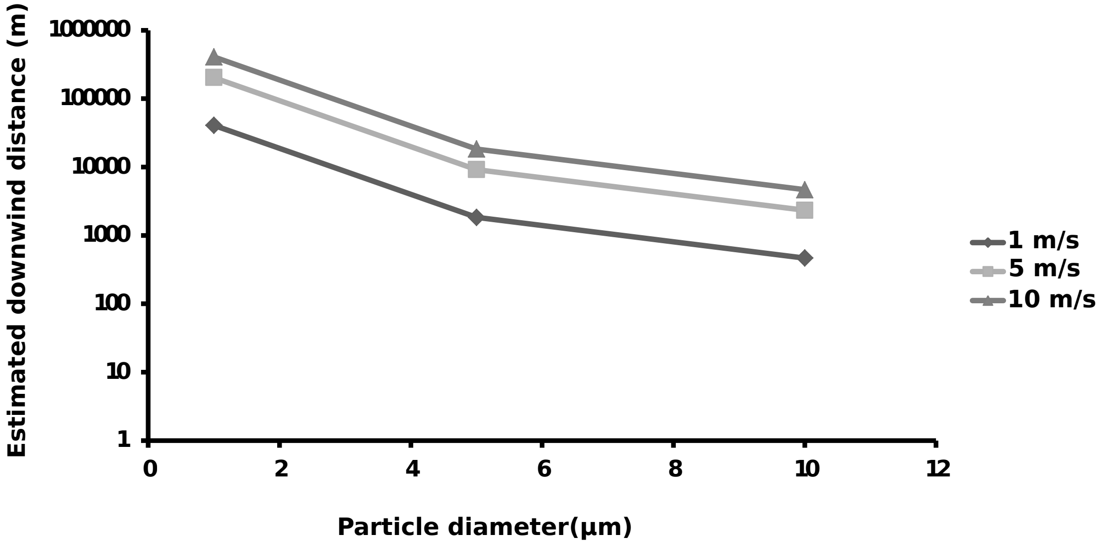

Supplement: Figure S2 — Estimation of distance traveled by a particle of 3 different sizes and under 3 different wind speeds. Assumptions: (1) Laminar air flow with no turbulence to disrupt the path of the particle; (2) A flat surface with no obstacles; (3) Horizontal velocity of the particle equal to wind speed; and (4) Vertical velocity equal to the terminal velocity of the particle in air. [file peerj-04-2827-s003.pdf]
